# Computational models in directed cell migration

**DOI:** 10.3389/fcell.2026.1815999

**Published:** 2026-05-07

**Authors:** Pablo Sáez

**Affiliations:** 1 Laboratori de Cacúl Numéric (LaCáN), Universitat Politécnica de Catalunya-BarcelonaTech., Barcelona, Spain; 2 IMTech (Institute of Mathematics), Universitat Politécnica de Catalunya-BarcelonaTech., Barcelona, Spain

**Keywords:** cell migration modeling, cell polarity, directed cell migration, durotaxis, electrotaxis

## Abstract

Directed cell migration is a fundamental biological process underlying development, tissue homeostasis, immune responses, and disease progression. While chemotaxis has long dominated conceptual frameworks of guidance, it is now clear that cells also respond robustly to physical cues such as mechanical stiffness gradients and electric fields. Still, how cells integrate multiple coexisting signals is poorly understood. Advances in experimental techniques have enabled precise control of these cues and revealed a rich diversity of taxis behaviors across cell types and environments. However, this experimental progress has outpaced the development of unifying theoretical frameworks capable of integrating multiple guidance modalities. In this review, we synthesize current understanding of well-known taxis, situating them within the broader landscape of physical taxis and highlighting common mechanistic themes. We discuss recent biophysical and computational models that aim to capture directed migration as an emergent property of coupled force generation, adhesion dynamics, and polarity regulation. Finally, we identify key experimental and theoretical gaps, and argue that integrated, multiscale modeling approaches are essential for moving from phenomenological descriptions toward predictive theories of cell migration in complex physiological settings.

## 1 Introduction

Directed cell migration (Majumdar et al., 2014; SenGupta et al., 2021) has been traditionally framed in terms of chemical gradients (Van Haastert and Devreotes, 2004), yet, over the past 3 decades, it has become clear that other physical signals can act as primary guidance cues for directed migration, reflecting the fundamentally multimodal nature of directed cell migration *in vivo* (Rørth, 2011; Lara Rodriguez and Schneider, 2013; Shellard and Mayor, 2019; Tetrick et al., 2025).

Among those tactic cues that rely on topological or material properties of the Extracellular Matrix (ECM), durotaxis (Sunyer and Trepat, 2020; Espina et al., 2022) has been largely studied. Durotaxis represents the tendency of cells to migrate along gradients of substrate rigidity, most commonly toward stiffer regions (Lo et al., 2000; Sunyer et al., 2016), but also toward softer ones (Koser et al., 2016; Isomursu et al., 2022). Early experiments demonstrated that substrate compliance modulates focal adhesion size, cytoskeletal organization, and migration speed, establishing matrix stiffness as an instructive signal (Pelham and Wang, 1997; Gallant et al., 2005; Engler et al., 2006; Ghassemi et al., 2012; Plotnikov et al., 2012; Schoen et al., 2013; Changede et al., 2015). At a mechanistic level, durotaxis is commonly understood through the interplay between actomyosin contractility, integrin-mediated adhesion (Vicente-Manzanares et al., 2009; Parsons et al., 2010; Kechagia et al., 2019), and force-dependent adhesion reinforcement (Chan and Odde, 2008; Elosegui-Artola et al., 2014; Oria et al., 2017): on stiffer substrates, forces are transmitted more effectively, leading to adhesion maturation and stabilized protrusions, which in turn bias cell polarity and persistence. While this picture is well supported in two-dimensional *in vitro* systems, its extension to three-dimensional, fibrous, and dynamically remodeling matrices remains an active area of investigation (Jacquemet et al., 2015; Yamada and Sixt, 2019; Saraswathibhatla et al., 2023).

There are other signals related to topological or material properties of the ECM. Cells can also migrate along gradients in time-dependent material properties (Liebchen et al., 2018; Shirke et al., 2021), a phenomenon termed viscotaxis, highlighting sensitivity not only to stiffness but also to matrix relaxation dynamics (Chaudhuri et al., 2015; Elosegui-Artola, 2021). Haptotaxis describes migration along gradients of substrate-bound ligands, such as ECM proteins (Carter, 1967; Fortunato et al., 2025), and was originally distinguished from chemotaxis by the immobilized nature of the cue, emphasizing the role of spatially patterned adhesion rather than soluble signals. Contact guidance or topotaxis arises from anisotropic environmental features, including aligned fibers, grooves, or ridges, which bias cell orientation and persistence through cytoskeletal alignment and directional adhesion formation (Park et al., 2018). Similarly, cells can migrate guided by cell-scale curvature variations (Pieuchot et al., 2018). In confined three-dimensional environments, cells may display barotaxis, preferentially migrating along paths of lower hydraulic resistance or pressure gradients, a behavior particularly relevant in complex tissue architectures (Polacheck et al., 2011; Paul et al., 2016; Lennon-Duménil and Moreau, 2021).

All these signals, related to properties of the ECM, share the same mechanistic nature as durotaxis: a tight coupling between polarization of the actomyosin network and integrin-mediated, force-dependent adhesion. There is another mode of directed cell migration that does not directly depend on integrin-based adhesion. Frictiotaxis (Shellard et al., 2025), the directed cell migration along gradients of external friction represents an adhesion-independent version of durotaxis. However, they still share one common feature: the friction is transmitted intracellularly, which, as in previous cues, finally induces an asymmetry in the retrograde flow. Therefore, we can group all those tactic cues as mechanotaxis because all these features of the ECM have a direct impact on the polarization of the actomyosin network and, eventually, on the mechanics of the retrograde flow (Roca-Cusachs et al., 2013; Mogilner and Manhart, 2018).

Another signal, which shares common mechanisms with chemotaxis, is electrotaxis, often also referred to as galvanotaxis. Electrotaxis describes directed migration in response to electric fields (EFs) (Cortese et al., 2014). Physiological EFs arise naturally *in vivo*, most prominently at epithelial wounds where disruption of transepithelial ion transport generates steady fields that persist until closure (Zhao et al., 2006; Zhao, 2009; McCaig et al., 2009). Many cell types–including epithelial cells, fibroblasts, keratinocytes, and immune cells–exhibit robust directional migration in EFs of physiological strength (Fang et al., 1999; Zhao et al., 1999; Zhao et al., 2002; Zhao et al., 2014; Sun et al., 2018; Zhu et al., 2019; Zhao et al., 2004; Pu et al., 2007; Wu et al., 2013). Unlike mechanotaxis, where force transmission provides an intuitive sensing mechanism, the means by which cells detect and transduce EFs remains more heterogeneous. Proposed mechanisms include asymmetric ion fluxes and calcium signaling (Shanley et al., 2006; Guo et al., 2015; Allen et al., 2013), electrophoretic redistribution of charged membrane proteins (Allen et al., 2013; Poo, 1981), polarization of signaling pathways such as PI3K/PTEN (Zhao et al., 2006), and cross-talk with growth factor receptors (Zhao et al., 2002). Indeed, electrotaxis often acts at the level of cell polarity establishment, mostly at the Rho GTPases signaling network (Ridley, 2015; Devreotes et al., 2017).

Cellular taxis mechanisms operate across physiological and pathological settings, and their relative contribution is best understood by considering the dominant microenvironmental cues in each condition. In physiological processes such as wound healing, chemotaxis driven by gradients of growth factors and cytokines (e.g., PDGF, TGF- β ) coordinates the early recruitment of immune cells and fibroblasts, while haptotaxis and durotaxis become increasingly relevant during matrix deposition and remodeling, where cells respond to ECM-bound ligands and stiffness gradients, respectively (Friedl and Gilmour, 2009). In fibrosis, persistent activation of fibroblasts and myofibroblasts is tightly linked to durotactic reinforcement, as progressive matrix stiffening amplifies mechanosensitive migration. In cancer, tumor cell invasion reflects a convergence of taxis mechanisms: chemotaxis toward soluble factors, haptotaxis along ECM gradients, and durotaxis toward stiffer stromal regions all contribute to metastatic dissemination, although much of the mechanistic insight derives from *in vitro* and engineered matrix systems, highlighting challenges in translating these findings *in vivo* (Yamada and Sixt, 2019; Pickup et al., 2014). Electrotaxis, while less explored in cancer, has been most robustly characterized in wound healing contexts where endogenous electric fields guide epithelial cell migration, suggesting potential but underutilized therapeutic relevance (Zhao et al., 2006). Overall, aligning each taxis modality with the dominant biophysical and biochemical features of specific disease contexts may improve the translational success of strategies aiming to modulate cell migration.

To date, much of the progress in understanding directed cell migration has been driven by experimental advances enabled by increasingly sophisticated control of chemical, mechanical, and electrical cues. While these studies have revealed a rich diversity of taxis behaviors, they have also exposed substantial variability across cell types and environments, underscoring the difficulty of isolating dominant mechanisms from experiments alone. Biophysical and computational models appear essential for distilling the minimal principles underlying taxis and for generating predictive hypotheses that extend beyond specific experimental systems. In particular, these approaches provide a natural framework for addressing how multiple guidance cues are integrated and for identifying unifying principles underlying apparently diverse taxis behaviors. This mini-review focuses on current modeling efforts in this area and outlines key challenges and future directions.

## 2 Biophysical ingredients in modelling taxis

**FIGURE 1:**
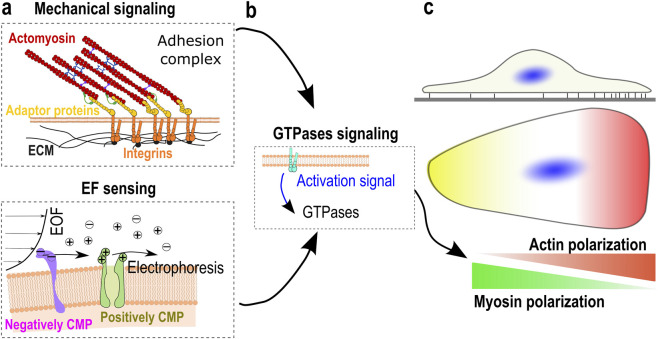
Main processes contributing to taxis. **(a)** First, dedicated proteins feel external cues, including mechanical, which are mostly transduced intracellularly by integrins through adhesion complexes and electrical signals, which are sensed by several transmembrane proteins, similarly to chemical cues. Adapted from (Venturini, 2023), licensed under CC BY 4.0. **(b)** Most of those proteins induce the activation of rhoGTPases, which cause **(c)** a polarization of the actomyosin network.

A central contribution in cell migration modeling has been to frame taxis not as a sequence of discrete sensing and decision-making steps, but as an emergent property of coupled physical processes. This shift has been important because it moves the interpretation of directional migration away from the notion of dedicated cellular “decision units” and toward a view in which directionality arises continuously from the interplay between force generation, adhesion dynamics, polarity regulation, and environmental constraints. Within this framework, cells do not need to explicitly compute gradients or select directions; instead, small asymmetries introduced by external cues are amplified through feedback between mechanics and intracellular organization, leading to persistent biased motion (see Figure 1). By grounding taxis in measurable physical interactions rather than abstract decision rules, this modeling viewpoint strengthens the connection between theory and experiment, and reshapes how molecular asymmetries observed in taxis assays are interpreted–not solely as sensors, but often as dynamic consequences of mechanically driven symmetry breaking.

In the context of durotaxis, most models have relied on phenomenological approaches, without an explicit feedback from cell adhesion (Novikova et al., 2017; Yu et al., 2017). Biophysical models have further shown that durotaxis can emerge from symmetry breaking driven by force-dependent adhesion maturation and contractility gradients (Copos et al., 2017; Rens and Merks, 2020; Betorz et al., 2023). One insightful class of models builds on the molecular clutch framework (Chan and Odde, 2008; Elosegui-Artola et al., 2014; Venturini and Sáez, 2023), in which actin retrograde flow, adhesion binding kinetics, and substrate stiffness jointly determine traction force transmission. When extended spatially, these models naturally produce directional biases in protrusion stabilization on stiffness gradients (Sáez and Venturini, 2023), without invoking an explicit stiffness sensor. Most existing models remain calibrated to linear two-dimensional elastic substrates; however they rarely include viscoelasticity (Chaudhuri et al., 2020; Shu and Kaplan, 2023; Saez et al., 2025), matrix degradation (Cicconofri et al., 2024), fiber alignment (Ahmadzadeh et al., 2017; Nahum et al., 2023; Ranamukhaarachchi et al., 2025; Chepizhko et al., 2025), and three-dimensional ECMs (Chaudhuri et al., 2016; Kim et al., 2018; Yamada et al., 2022).

Electrotaxis modeling has followed a somewhat different trajectory, reflecting the diversity of proposed sensing mechanisms and the shared signaling mechanisms with chemotaxis. Early phenomenological models treated EFs as external biases acting on cell polarity vectors (Erickson et al., 2001), which usually only report cell velocity trends without dynamic intracellular data (Vanegas-Acosta et al., 2012). More mechanistic approaches have incorporated electrodiffusion and electrophoresis of membrane-bound signaling components (Allen et al., 2013; McLaughlin and Poo, 1981; Sarkar et al., 2019), showing how small fields can generate intracellular asymmetries over experimentally relevant timescales. While these frameworks successfully reproduce directional bias and threshold effects observed experimentally, they often remain decoupled from cell mechanics, treating migration again as a polarity-driven process rather than a force-generating one. As a result, the interaction between EFs and the motility machinery of the cell remains mostly underexplored in theoretical work. Recently, integrated active gel, sensing, and signaling models have led to an holistic approach where the complete chain of events leads to electrotaxis (Kulkarni et al., 2025). Importantly, the modeling of ion-channel-mediated dynamics and their coupling to actin regulation has been poorly analyzed (Gao et al., 2011; Zhang and Cui, 2015; Tsai et al., 2020), which should be further explored in the future.

Taken together, these insights reveal minimal principles to predict hypotheses that extend beyond specific experimental tactic systems. In particular, they provide a natural framework for addressing how multiple guidance cues are integrated and for identifying unifying principles underlying apparently diverse taxis behaviors (Betorz et al., 2023; Saez et al., 2025; Yang et al., 2021; Sáez et al., 2026).

## 3 Computational approaches and their alignment with experimental assays

Computational approaches for cell migration also play a central role in our understanding of directed cell migration. The suitability of a given modeling framework to address taxis depends not only on its spatial resolution, but also on whether taxis is assumed to arise from explicit gradient sensing at the single-cell level or as an emergent property of multicellular coupling. And, naturally, this is directly aligned with the outcomes of the experimental assays.

Experimental assays for taxis span a wide range of spatial and organizational scales, from isolated single cells exposed to well-controlled gradients to extended multicellular tissues migrating in complex environments. Modeling frameworks differ substantially in how naturally they connect to these experimental paradigms, and this alignment strongly shapes how taxis mechanisms are interpreted. A summary of the following approaches is presented in Table 1.

**TABLE 1:** Comparison of computational approaches for modeling taxis across experimental contexts.

Model class	Taxis mechanisms modeled	Experimental context best represented	Key assumptions/representation	Strengths	Limitations/gaps
Particle-based models	Primarily chemotaxis; generic directional biases (incl. duro-/electro-taxis phenomenologically)	Single-cell tracking assays; collective migration with coarse-grained readouts (e.g., streams, invasion fronts)	Cells as self-propelled particles with velocity/polarity updates; taxis as external bias or alignment rule	Captures emergence of collective taxis from simple rules; robust to noise; directly matches trajectory statistics	No explicit cell shape, forces, or intracellular dynamics; cues imposed phenomenologically; limited mechanistic insight
Lattice-based (CPM)	Chemotaxis; some mechanotaxis/electrotaxis via energy biases	Single-cell and multicellular assays with shape changes (e.g., clusters, wound closure, spheroids)	Cells as deformable domains on lattice; taxis as effective energy term biasing protrusions or polarity	Includes cell shape, adhesion, and multicellular rearrangements; suitable for collective amplification of weak cues	Intracellular mechanics and force balance implicit; limited resolution of traction forces; phenomenological signaling
Vertex/Voronoi models	Indirect taxis via tissue mechanics; collective guidance	Confluent epithelial systems; wound healing assays; monolayer expansion	Cells represented by polygons; mechanics dominated by junctional tension and area constraints	Ideal for tissue-scale studiess, jamming/fluidity, and collective guidance	Poor representation of single-cell sensing and intracellular detail
Continuum/active gel models for single-cell	Mechanotaxis, electrotaxis, chemotaxis (explicit coupling to signaling and forces)	High-resolution single-cell assays (traction force microscopy, micropatterns, stiffness gradients, electric fields)	Continuous fields for actin, myosin, adhesion, signaling; explicit force balance	Direct link to measurable quantities (forces, polarity, signaling); mechanistic description of cell migration	Computationally intensive; parameter-rich; difficult to scale to large tissues
Phase-field	Mechanotaxis, chemotaxis; emergent collective taxis	Single-cell and collective assays with deformable boundaries; tissue spreading, invasion	Diffuse interface for cell boundary; coupled chemical/mechanical fields; active stresses	Captures shape and force generation; can bridge single-cell to tissue scale	High computational cost; parameter calibration
Immersed Boundary Methods (IBM)	All taxis with explicit mechanotransduction modeling	2D/3D environments with complex geometry; cells in ECM or fluid	Elastic structures (membrane, cortex) coupled to viscous environment	Handles complex geometries; realistic force transmission	Very computationally demanding; dlimited large-scale applications
Continuum tissue-scale models	Collective taxis (emergent from mechanical coupling)	Tissue-level assays (wound healing, monolayers, expanding tissues)	Coarse-grained fields (density, velocity, stress); weak biases amplified via coupling	Captures long-range coordination, stress propagation, and collective guidance	Lacks single-cell resolution; mechanistic interpretation of sensing is indirect
Data-driven/ML/inference models	Any taxis (learned from data)	Trajectory datasets from diverse assays (single-cell and collective)	Statistical or neural models trained on migration data; no explicit physics required	High predictive power; integrates multiple datasets; useful for control/optimization	Limited interpretability; mechanisms not explicitly analyzed; poor extrapolation

Particle-based models have been widely used to study taxis across both single-cell and collective regimes (Vicsek et al., 1995; Szabó et al., 2006; Van Liedekerke et al., 2015), particularly when the focus lies on emergent alignment, long-range coordination, or robustness to noise. Taxis can be incorporated as directional biases acting on cell polarity or velocity, making these models ideal for exploring how different guidance cues compete or cooperate at the population level. In collective migration, particle models have been instrumental in demonstrating how taxis can arise even when individual gradient sensing is weak or absent, provided that alignment and interaction rules are sufficiently strong.

Lattice-based models such as the Cellular Potts Model (CPM) (Graner and Glazier, 1992; Glazier and Graner, 1993) have also been extensively used to study chemotaxis and, to a lesser extent, mechanotaxis and electrotaxis, in both single-cell and collective contexts (Rens and Merks, 2020; Chen et al., 2007; Allena et al., 2016). Because cues are usually incorporated as effective energy biases, favoring polarity alignment, CPMs naturally lend themselves to phenomenological descriptions of guidance. In collective settings, this allows one to explore how weak single-cell biases are amplified through cell-cell adhesion and shape coupling, leading to coherent directed motion of clusters or sheets.

Experimental assays that quantify directional persistence, turning-angle distributions, and net population drift, at both the single-cell and collective levels, map naturally onto particle-based models. These assays yield coarse-grained variables such as migration speed, persistence time, and bias along imposed cues, which correspond directly to state variables or control parameters in these frameworks. In both particle-based and CPM approaches, external fields or gradients are implemented phenomenologically as effective biases on cell polarity, protrusion dynamics, velocity updates, or cell-cell interactions, enabling direct calibration against experimentally measured migration statistics and making these models well-suited for testing hypotheses related to noisy cue sensing, adaptation, and stochastic fluctuations in directional decision-making. While CPMs explicitly represent cell shape, adhesion, and tissue-level rearrangements, they do not resolve intracellular force balance or spatiotemporally evolving fields, such that links between guidance cues and intracellular or intercellular stress redistribution remain implicit rather than emergent. Consequently, these models are most informative for assays reporting coarse-grained migratory outcomes, such as net drift, persistence, or collective alignment, but are less suited for dissecting taxis mechanisms that depend sensitively on intracellular force transmission, adhesion turnover, or traction asymmetries, as is the case in taxis.

Vertex and Voronoi formulations have been especially influential in epithelial systems, where collective migration and taxis are tightly constrained by confluency (Fletcher et al., 2014; Alt et al., 2017). These models are well-suited to addressing guidance mechanisms that operate through tissue-scale geometry and junctional mechanics. Vertex-based models are most effective for understanding how collective constraints reshape taxis responses, rather than how individual cells sense gradients, because cell shape and internal force generation are treated in a highly reduced manner.

When the experimental focus shifts to detailed single-cell dynamics, continuum models become particularly powerful (Mogilner and Manhart, 2018; Prost et al., 2015; Danuser et al., 2013). These approaches explicitly resolve spatial and temporal variations in molecular polarization, actomyosin organization, adhesion kinetics, and traction force generation, enabling direct comparison with high-resolution experimental measurements of protrusion-retraction dynamics, force distributions, and intracellular signaling patterns. Single-cell mechanotaxis assays, such as migration on stiffness gradients or micropillar arrays, are therefore most naturally interpreted using continuum frameworks that incorporate substrate mechanics, force-dependent adhesion maturation, and asymmetric force transmission (Sáez and Venturini, 2023). Molecular sensing processes across spatial scales, observed in durotaxis, can also be encoded phenomenologically and linked to continuum parameters (Sáez and Venturini, 2023; Kulkarni et al., 2025).

Taxis assays align well with continuum descriptions. These assays are typically performed on isolated cells exposed to uniform DC EFs or mechanical gradients, and are often accompanied by detailed measurements of membrane protein and adhesion polarization, cytoskeletal reorganization, and intracellular signaling redistribution (Zhao et al., 2002; Zhao et al., 2014; Wu et al., 2013). Continuum models can capture how electric fields bias actomyosin organization and polarity through electromechanical coupling, leading to symmetry breaking and directed migration.

Continuum models are discretized numerically in space through several techniques, from the standard Finite Element Method to more advanced approaches to describe moving boundaries. Phase-field models (Moure and Gomez, 2021; Kuang et al., 2023) allow a direct representation of the moving cell boundary and the interaction between the cell and its environment (Nonomura, 2012; Ziebert and Aranson, 2013; Marth and Voigt, 2014). Phase-field and active-gel formulations naturally incorporate force balance, actin-driven protrusion, and adhesion (Shao et al., 2012). In collective migration (Nam et al., 2016; Alert and Trepat, 2020), these models naturally capture how taxis at the tissue scale can emerge from mechanical coupling, stress transmission, and competition between cell-cell and cell-substrate interactions (Moure and Gomez, 2021). Immersed Boundary Methods (IBM) are perfect candidates to describe complex shape changes in 2D and 3D (Rejniak, 2007). The IBM, originally developed to study fluid-structure interactions (Peskin, 2002; Mittal and Iaccarino, 2005), has been adapted to cell motility (Liu et al., 2006; Stryc et al., 2015) by explicitly coupling actin-driven protrusive forces, cortical tension, and adhesion-mediated traction to the surrounding viscous cytoplasm and ECM. Looking forward, and given the current computational power and the future to come, hybrid multiscale approaches, combining continuum descriptions of chemical, mechanical, or electrical fields resolved by the IBM, offer a promising route to incorporate all the physical aspects in the modeling of single and collective directed cell migration, and unify multiple taxis within a single framework.

At larger spatial scales, where experimental resolution shifts from intracellular details to tissue-level coordination, collective taxis assays (Sunyer et al., 2016; Cohen et al., 2014; Zajdel et al., 2020; Lacroix et al., 2024) are particularly well matched to continuum models (Alert and Trepat, 2020). Continuum descriptions are well-suited to assays probing robustness and long-range coordination, where individual cell-level details are experimentally less accessible. However, these frameworks can also be adapted to capture how weak directional biases at the single-cell level are amplified through mechanical coupling and signaling feedback across scales, while capturing rich intracellular information (Wörthmüller et al., 2025). Wound-healing assays and expanding epithelial monolayers, which emphasize the role of cell-cell junctions, tissue fluidity, and collective force transmission, align naturally with vertex and Voronoi models that explicitly represent junctional tension, neighbor exchanges, and large-scale tissue mechanics (Alt et al., 2017; Ouzeri et al., 2025).

Although experiments robustly demonstrate directional migration specifically in applied EFs, but also in mechanical and chemical gradients, the underlying sensing and signaling mechanisms remain incompletely resolved. Consequently, recent modeling efforts have increasingly relied on data-driven descriptions of cell migration (Brückner and Broedersz, 2024; Sargent et al., 2022; Baker et al., 2025), including neural-network-based models (Marquez et al., 2025) and Bayesian inference frameworks (Prescott et al., 2021), which are trained directly on trajectory data, as well as optimal control theory to analyze strategies for steering electrotactic responses in space and time (Martina-Perez et al., 2024). These approaches can successfully reproduce observed migration statistics and predict responses to perturbations, but they typically remain phenomenological: the learned rules operate at the level of effective behaviors rather than being expressed in terms of directly measurable mechanical forces or biochemical signaling components.

## 4 Discussion

The growing recognition of a multitude of physiologically relevant guidance mechanisms has fundamentally expanded the conceptual landscape of directed cell migration (SenGupta et al., 2021). These behaviors underscore that migration is not governed by a single dominant cue, but rather emerges from the continuous integration of chemical, mechanical, and electrical signals. Mechanotaxis and electrotaxis represent two paradigmatic examples in which cells respond directionally to spatial gradients. Despite their distinct physical origins, these taxis emerge from the integration of multiple chemical and physical mechanisms, force generation, adhesion dynamics, membrane polarization, and ion transport, that convert weak external gradients into persistent directional motion through internally amplified feedback loops whose relative influence depends on cellular state and environmental context.

Experimental studies have been instrumental in revealing the existence, robustness, and diversity of these responses in isolation, yet they also expose the context dependence of taxis, with outcomes that vary across cell types, dimensionality, and environmental architecture. This variability challenges purely phenomenological interpretations and motivates the search for underlying principles that transcend specific experimental implementations. Furthermore, if several cues coexist *in vivo* with bio-electro-chemical gradients, topographical and mechanical features, it raises the question of how cells integrate multiple, sometimes competing, guidance signals.

Guidance cues rarely act in isolation, but instead interact with each other to reinforce or compete during directed cell migration. In many systems, these cues act synergistically: for example, stiffness gradients can enhance chemotactic migration by stabilizing cell polarity and promoting adhesion maturation, thereby increasing directional persistence. Similarly, endogenous electric fields present in wound healing can bias intracellular signaling pathways (e.g., PI3K/PTEN) that are also central to chemotaxis, effectively aligning multiple guidance mechanisms toward a common direction. However, competing cues can also arise, particularly in heterogeneous environments such as tumors or fibrotic tissues, where conflicting gradients of soluble factors, matrix stiffness, and topography may lead to biased or even impaired directional migration. In such cases, cells must hierarchically integrate multiple inputs. Mechanical cues could dominate over chemical gradients when they strongly affect force transmission and adhesion dynamics, while chemotaxis may prevail in environments with weak or uniform mechanical structure. These interactions highlight that taxis should be understood as a multi-cue integration process rather than a single-cue response, and that the relative dominance of each guidance mechanism depends critically on the spatial and temporal organization of the microenvironment.

Biophysical and computational models provide a critical means to address this challenge. Most existing models remain specialized, neglecting key features of physiological environments, including three-dimensionality, matrix remodeling, and the coexistence of multiple, dynamically evolving cues. Another important emerging challenge, and opportunity, lies in developing integrated mechanochemical-bioelectrical models capable of addressing multiple taxis within a unified framework. *In vivo*, mechanical signals, EFs, chemical cues, and tissue-scale constraints are not isolated variables but dynamically coupled fields. Yet most current models focus on a single guidance cue in isolation. Looking forward, a central challenge for the field is the development of integrated frameworks capable of capturing cue competition, cooperation, and hierarchy in directed migration (Sáez and Venturini, 2023; Saez et al., 2025; Sáez et al., 2026). Further research directions include (i) explicit coupling between EFs and mechanosensitive ion channels or adhesion dynamics, (ii) multiscale links between subcellular polarization, traction-force generation, and tissue-level migration, and (iii) predictive criteria for cue dominance or switching when multiple gradients coexist.

Across all assays, another central conceptual lesson is that the same experimental observation–directed motion along a gradient–may arise from fundamentally different mechanisms depending on scale. Single-cell assays primarily probe sensing and intracellular polarization, whereas collective assays often probe amplification, force transmission, and emergent guidance (Friedl and Gilmour, 2009; Mayor and Etienne-Manneville, 2016). Models that explicitly match the assay scale are therefore essential for drawing mechanistic conclusions. A key aspect for future contributions will be to incorporate highly detailed models at the single-cell level into large-scale tissue using multiscale computational models.

Addressing these gaps will likely require combining continuum descriptions of multiple fields. Approaches that combine continuum descriptions of chemical, mechanical, and electrical fields with subcellular force- and molecular-resolving models are particularly promising in this regard. By explicitly linking environmental cues to intracellular polarity, force generation, and adhesion dynamics, models can reveal how weak external gradients are amplified into persistent directional motion. Such a framework is well positioned to move the field from qualitative explanations of taxis toward quantitative, testable predictions and to provide a common language connecting different forms of directed migration. Such models are also well positioned to bridge single-cell and collective behaviors, to generate experimentally testable predictions, and to guide the design of perturbations that selectively probe dominant mechanisms.

Ultimately, progress in understanding taxis will depend on a tighter coupling between quantitative experiments and theory, which will clearly benefit from data-driven approaches (Brückner and Broedersz, 2024; Baker et al., 2025; Marquez et al., 2025; Prescott et al., 2021; Martina-Perez et al., 2024). Data-driven approaches can be not only used to rationalize observed behaviors, but also to predict when, where, and how cells choose one guidance cue over another in complex biological contexts.
